# Purpurin Rescues Contrast-Induced Acute Rat Kidney Injury via Inducing Autophagy and Inhibiting Apoptosis

**DOI:** 10.3390/ph19010116

**Published:** 2026-01-08

**Authors:** Kangxu He, Xiaoying Sun, Xinhui Pan, Xiaoda Yang, Qi Wang, Kai Liao

**Affiliations:** 1 Key Laboratory of Xinjiang Phytomedicine Resource and Utilization, Ministry of Education, Institute for Safflower Industry Research, School of Pharmacy, Shihezi University, Shihezi 832002, China; hkx16699040910@163.com (K.H.); s17731511105@163.com (X.S.); panxhshzu@shzu.edu.cn (X.P.); xyang@bjmu.edu.cn (X.Y.); 2 Stake Key Laboratory of Natural and Biomimetic Drugs, Department of Chemical Biology, School of Pharmaceutical Sciences, Peking University, Beijing 100191, China

**Keywords:** contrast-induced acute kidney injury, purpurin, antioxidant, apoptosis, autophagy, rat

## Abstract

**Objectives**: Contrast-induced acute kidney injury (CIAKI) is a major cause of hospital-acquired renal injury, and strategies for its treatment are currently lacking. This study aimed to investigate the amelioration effect and mechanism of purpurin, a natural antioxidant, against CIAKI via an integrated analysis of network pharmacology, bioinformatics, molecular docking, and animal experiments. **Methods:** Network pharmacology approaches were used to predict key targets of purpurin against CIAKI. The differential expression of these key targets was further investigated using bioinformatics analysis and molecular binding with purpurin by molecular docking. A CIAKI model was established in SD rats via iohexol administration, and they were treated with 2.5 mg/kg or 5 mg/kg purpurin. Related physiological and pathological indexes were detected to explore the intervention mechanism. **Results:** Key gene targets were screened from protein–protein interaction networks, of which Pik3c2a, Esr1, Aktip, HSP90AA1, Bcl2, Caspase3, and SRC in the CIAKI group of GSE189881 were significantly differentially expressed compared to the control group. Molecular docking results show that PI3K, ESR1, HSP90, CASP3, AKTI, and SRC had the highest level of connectivity with purpurin. In vivo experiments demonstrated that the Scr and BUN increased in CIAKI rats, the pathological morphology of renal tissue deteriorated, the levels of TNF-α, IL-1β, and IL-6 increased, the contents of MOD and NO in oxidative stress increased, and the activity of SOD and GSH-PX decreased. After administration of purpurin, the above indexes improved in a dose-dependent manner (<0.05). Western blotting showed that purpurin inhibited the Beclin1/Bcl-2/caspase-3 apoptotic cascade and induced the P62/LC3 autophagy pathway. **Conclusions:** This study provides experimental evidence supporting purpurin as a potential therapeutic agent for CIAKI and further explores its antioxidant mechanisms.

## 1. Introduction

Contrast-induced acute kidney injury (CIAKI) is defined as acute impairment of renal function occurring within 24 to 72 h following the administration of iodinated contrast media (ICM) [1]. As one of the leading causes of hospital-acquired renal failure, CIAKI may precipitate the progression from acute kidney disease to chronic kidney disease and even to end-stage renal disease [2,3]. However, current strategies for the prevention and management of CIAKI remain limited. Beyond intravenous hydration, many international clinical practice guidelines currently lack recommendations on widely applicable medicine interventions [4,5]. Thus, the development and validation of effective agents against CIAKI represent a critical challenge.

The pathophysiological mechanisms underlying CIAKI are complex and remain incompletely elucidated. Current studies identify the cytotoxicity and renal ischemia–hypoxia induced by ICM as central pathogenic drivers [6]. When ICM are exposed to renal tissue, specifically renal tubular epithelial cells (RTECs), they may trigger mitochondrial dysfunction-induced reactive oxygen species (ROS) generation, which further promotes oxidative stress and an inflammatory response, leading to a malignant progression that amplifies renal damage [7,8,9,10]. Consequently, current studies showed that targeting ROS overproduction through the protection of mitochondrial function, enhancement of peroxide clearance, or upregulation of endogenous antioxidants can effectively alleviate the pathological progression of CIAKI, thereby exerting antioxidant, anti-inflammatory, and anti-apoptotic effects [11,12,13,14].

Purpurin, a principal active constituent in the roots of *Rubia cordifolia*, has demonstrated multiple pharmacological activities [15]. It possesses strong free radical scavenging ability in the DPPH assay [16], and reduces intracellular ROS generation by triggering the Nrf2 signaling axis, as shown in an in vitro study [17]. This evidence demonstrates the potent antioxidant effects of purpurin and its potential for intervening in CIAKI, although the therapeutic effects and relative pharmacological mechanisms are not completely clear.

In this study, network pharmacology prediction, bioinformatics microarray, and animal experimental validation analysis were integrated to investigate the therapeutic effects of purpurin against CIAKI and elucidate its molecular mechanisms. Our findings may further elucidate the antioxidant mechanism of purpurin and lay a theoretical foundation for its clinical translation as a candidate agent for CIAKI management. Therefore, the purpose of the current study is to evaluate the therapeutic effects and relative mechanisms of purpurin against CI-AKI based on an integrated strategy that combines network pharmacology prediction, a bioinformatics microarray, and experimental validation in animals. Notably, our study also aims to provide an in-depth explanation of the antioxidant mechanism of purpurin.

## 2. Results

### 2.1. Network Pharmacology Analysis

**Figure 1:**
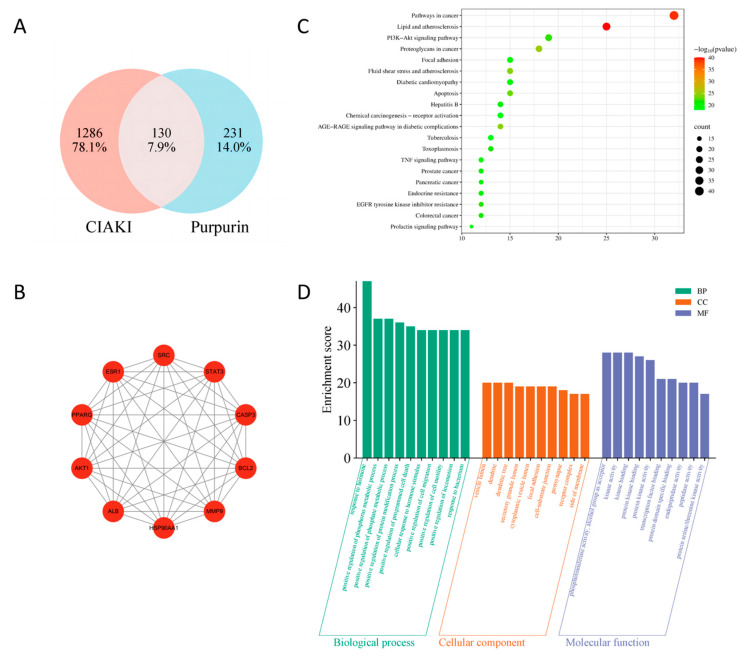
Target gene network construction and enrichment analysis. (**A**) Venn diagram of purpurin and CIAKI genes; (**B**) PPI network of the top 10 core target genes; (**C**) KEGG pathway enrichment analyses; (**D**) GO pathway enrichment analyses.

Network pharmacology prediction was performed to explore the potential target pathways of purpurin intervention on CIAKI. A total of 361 target genes for purpurin and 1416 CIAKI-related target genes were retrieved from corresponding databases. Venn diagram analysis revealed 130 common target genes between datasets (Figure 1A). PPI network analysis identified the top 10 core target genes (Figure 1B), and enrichment analysis was performed on common targets. KEGG analysis further revealed that these targets were significantly linked to pathways involving cancer, lipid and atherosclerosis, and to PI3K-Akt signaling pathways (Figure 1C). GO analysis demonstrated that significantly enriched biological processes (BPs) included hormones, positive regulation of the phosphorus metabolic process, and positive regulation of the phosphate metabolic process. Cellular components (CCs) included vesicle lumens, dendrites, and dendritic trees, whereas molecular functions (MFs) included phosphotransferase activity, alcohol groups as acceptors, kinase activity, and kinase binding (Figure 1D).

### 2.2. DEG Analysis

**Figure 2:**
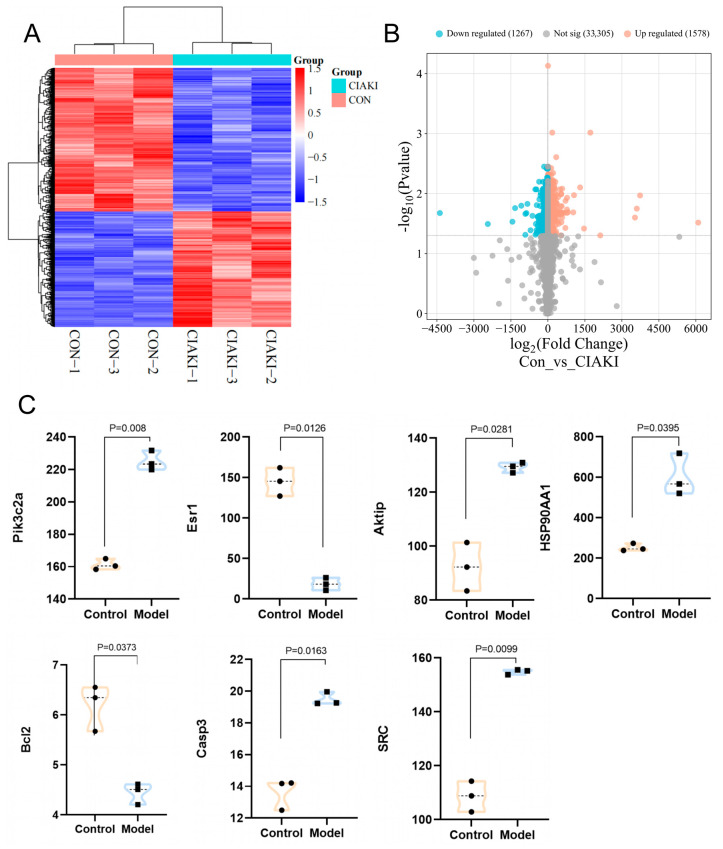
Screening of key target genes based on the CIAKI gene expression dataset GSE189881. (**A**) Heatmap; (**B**) volcano plot; (**C**) the gene expression of Pik3c2a, Esr1, Aktip, HSP90AA1, Bcl2, Casp3, and SRC.

RNA-Seq data were used to analyze the changes in gene expression differences between the control and CIAKI model groups. DEGs with statistical significance were screened using thresholds of |log2FC| ≥ 1 and *p* < 0.05. Volcano plot analysis revealed that the model group exhibited a total of 2854 DEGs compared to the control group, comprising 1578 upregulated and 1276 downregulated genes (Figure 2A,B). The expression of target genes that were identified in DEGs, and also PI3K/AKT-associated hub targets identified in network pharmacology analysis, was visualized in the datasets (Figure 2C). The results showed that the expressions of the Pik3c2a, Esr1, Aktip, HSP90AA1, Bcl2, Caspase3, and SRC genes in the CIAKI group were significantly different from those in the control group (*p* < 0.05), and the ALB and MMP9 genes showed no significant difference.

### 2.3. Purpurin Intervention in Molecule Docking in CIAKI

**Figure 3:**
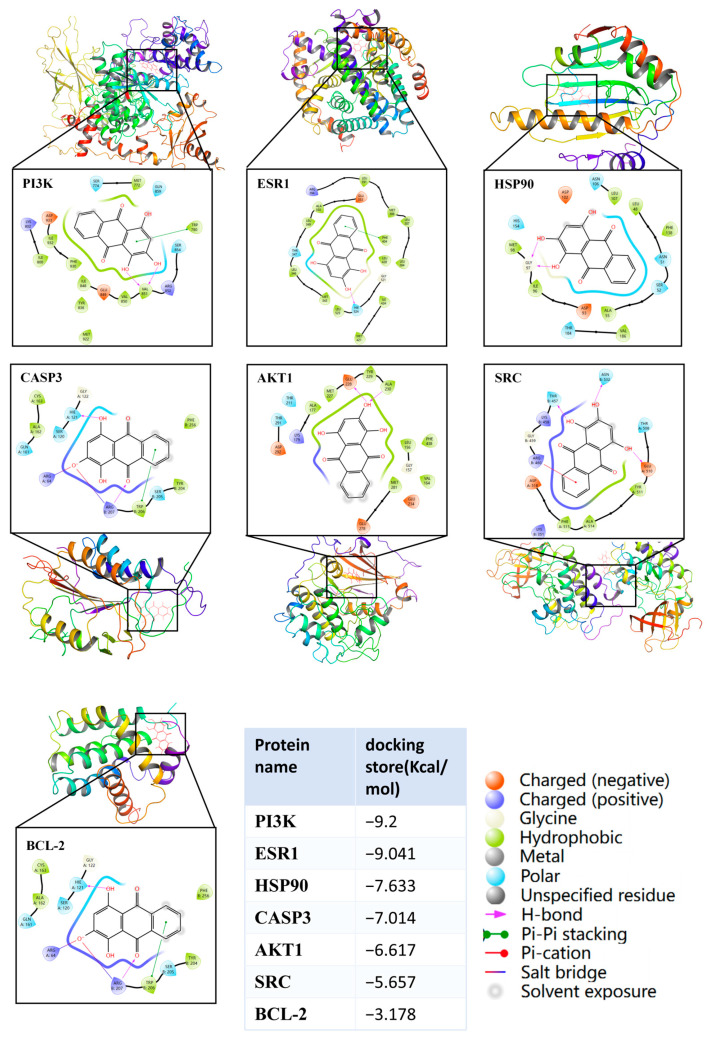
The molecular docking results of purpurin and core targets that interfere with CIAKI.

Molecular docking simulations were performed between purpurin’s chemical structure and the protein structure of the core targets identified through sequencing analysis and network pharmacology screening. The docking results demonstrated that the binding energies of PI3K, ESR1, HSP90, CASP3, AKTI, and SRC were less than −5 kJ/mol, except for BCL-2 (−3.178), indicating the formation of strong molecular interactions (Figure 3).

### 2.4. Purpurin Attenuates Contrast-Induced Renal Injury in SD Rats

**Figure 4:**
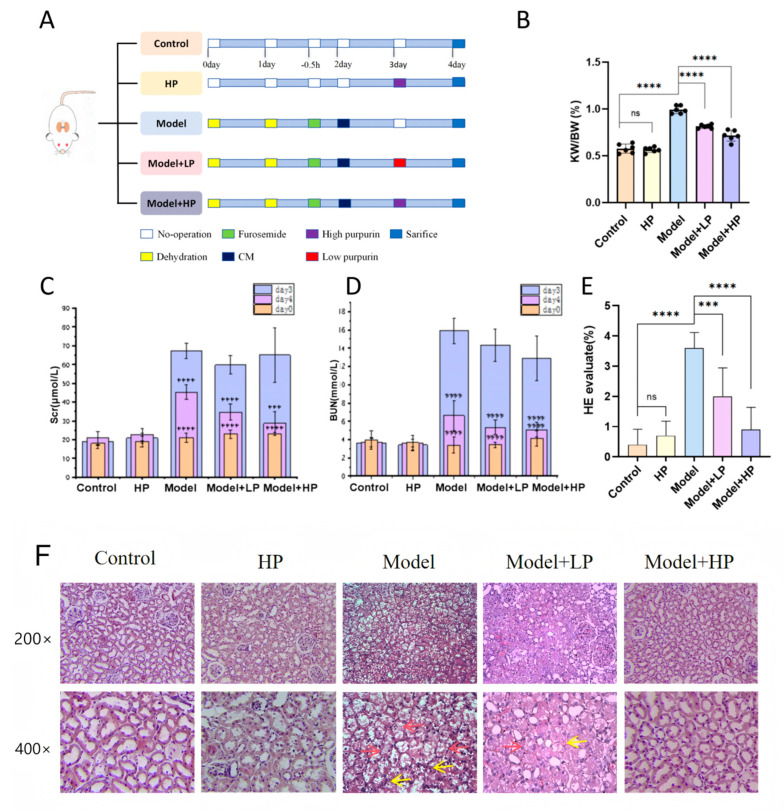

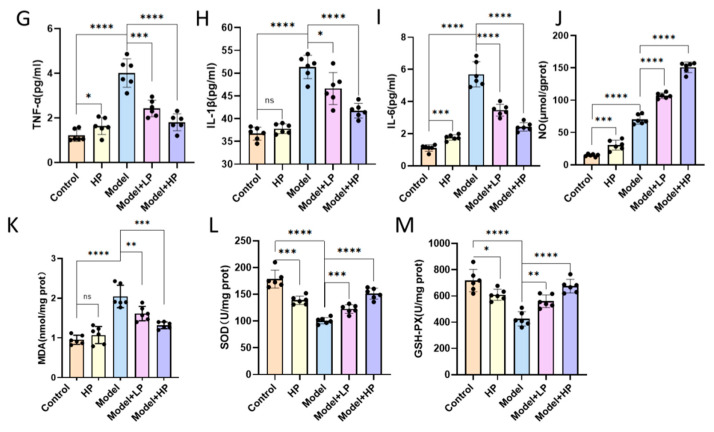
Biochemical Parameters Demonstrating the Amelioration of Purpurin on CIAKI. (**A**) Schematic diagram of the CIAKI SD rats model; (**B**) KW/BW; (**C**,**D**) Serum level of Scr and BUN; (**E**) Paller scoring of renal HE staining; (**F**) HE staining, the red arrow represents RTEC swelling, vacuolization, necrosis, and detachment, while the yellow arrow represents inflammatory cell infiltration within the renal tubules; (**G**–**L**) Serum level of TNF-α, IL-1β, IL-6; (**J**–**M**) The level of NO, MDA, SOD, GSH-PX in renal. Data were expressed as the mean ± SD, *n* = 6, ^ns^ *p* > 0.05, * *p* < 0.05, ** *p* < 0.01, *** *p* < 0.001, **** *p* < 0.0001.

We aimed to explore the efficacy of purpurin on CIAKI in vivo. The treatment regimen of purpurin for CIAKI rats is illustrated in Figure 4A. To monitor the kidney injury before and after modeling and drug intervention, creatinine (Scr) and blood urea nitrogen (BUN) levels were measured at three time points. On day 3, rats subjected to modeling showed significant elevations in Scr and BUN levels compared to the baseline (day 0), indicating successful establishment of the CIAKI model. Both the Model+LP and Model+HP groups exhibited significant reductions in Scr and BUN levels compared to the model group on day 4, indicating an improvement in renal function. In addition, no significant differences in Scr or BUN were observed between the control group and the HP group across the three time points (Figure 4C,D). Scr and BUN measurements and histological analysis of renal tissues in the model group demonstrated significant pathological alterations compared to controls, as evidenced by findings from HE staining. These included RTEC swelling, vacuolization, necrosis, and detachment, along with inflammatory cell infiltration within renal tubules (Figure 4E,F). In addition, the model group showed a significantly higher kidney-weight-to-body-weight (KW/BW) ratio compared with the control group (Figure 4B). These pathologic changes in AKI were markedly alleviated in purpurin-treated groups.

### 2.5. Purpurin Attenuates Contrast-Induced Oxidative Stress and Inflammation in SD Rats

The occurrence of oxidative stress and the release of inflammatory factors played important roles in the pathogenesis of CIAKI. ELISA analysis revealed significantly elevated levels of TNF-α, IL-1β, and IL-6 in the model group compared with the control groups, which were substantially attenuated in the Model+LP and Model+HP groups (Figure 4G–I). In addition, oxidative stress biomarkers in renal tissue revealed that the model group exhibited significantly increased MDA and NO levels alongside decreased SOD and GSH-Px activities compared with the control group. Compared with the model group, changes in MDA levels and GSH-Px and SOD activities were reversed by purpurin, but NO levels showed further increases (Figure 4J–M).

### 2.6. Purpurin Improves CIAKI-Induced Apoptosis in SD Rats

**Figure 5:**
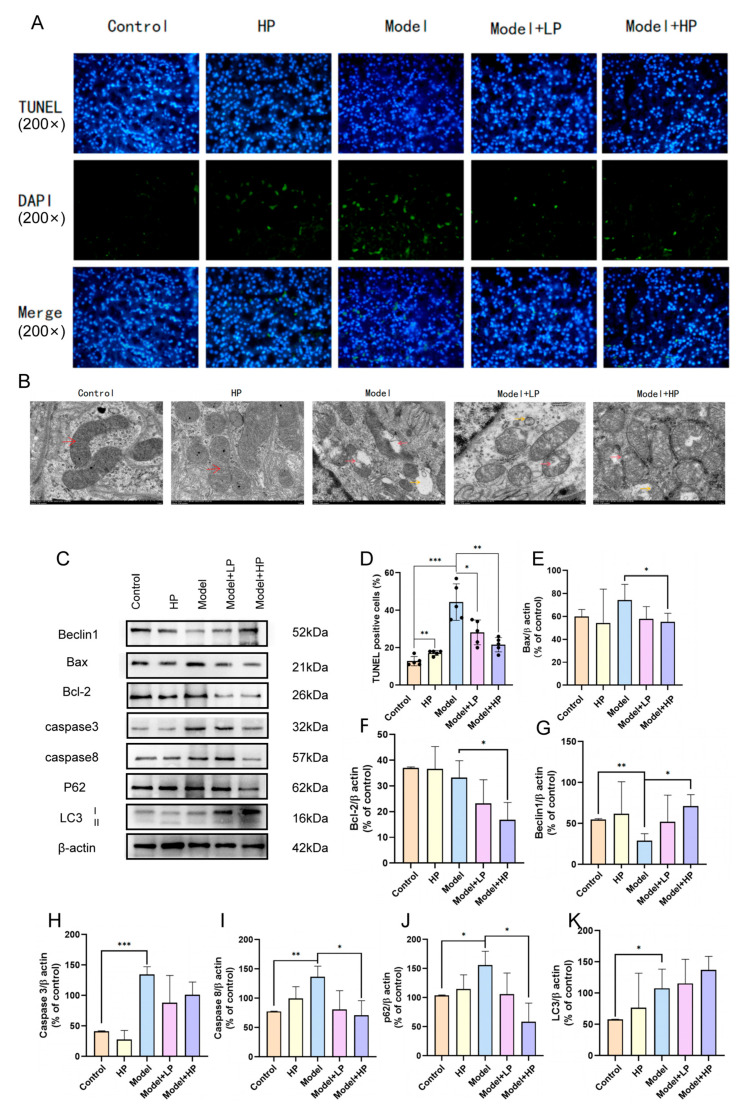
Purpurin improved apoptosis and inhibited autophagy in the CIAKI rat model. (**A**) TUNEL staining; (**B**) TEM images of mitochondria in RTECs, the yellow arrow represents the autophagosome, and the red arrow represents the mitochondrion; (**D**) Quantification of TUNEL-positive cells; (**C**,**E**–**K**) Western blot analysis and quantification of Bax, Bcl-2, Beclin1, Caspase-3, Caspase-8, p62, and LC3-II. Data were presented as mean ± SD. *n* = 5 (**A**,**D**), *n* = 3 (**E**–**K**) * *p* < 0.05, ** *p* < 0.01, *** *p* < 0.001.

TUNEL staining revealed a significantly increased number of apoptotic cells in the model group compared to the control group, while purpurin treatment markedly reduced cell apoptosis (Figure 5A,D). Transmission electron microscopy demonstrated that the mitochondrial morphology of RTECs in the control and HP groups was normal. The model group exhibited severe mitochondrial swelling, vacuolization, and disappearance of cristae structures, which were ameliorated in purpurin-treated groups (Figure 5B). The Western blot results indicated that purpurin modulated the expression of apoptosis-related proteins by upregulating Beclin1 and downregulating Bax and Bcl-2 (Figure 5E–G). It also regulated the expression of autophagy-related proteins through upregulating LC3-II and downregulating p62. In addition, purpurin treatment decreased the expression levels of Caspase-3 and Caspase-8, which were generally identified as the downstream of both apoptotic and autophagic pathways (Figure 5H,I).

## 3. Discussion

ICM are one of the most frequently utilized pharmaceuticals, with over 75 million clinical examinations employing it globally each year [18]. As a significant global public health concern, CIAKI is associated with increased morbidity, mortality, and healthcare costs. Although current mainstream iso- or low-osmolar contrast agents exhibit improved safety compared to earlier formulations, the dangers of CIAKI in high-risk populations should not be overlooked. Therefore, exploring effective preventive and therapeutic strategies for CIAKI carries significant clinical importance [2,19]. Purpurin, a key active constituent first extracted from the roots of *Rubia cordifolia* L., has demonstrated protective effects in various tissue injury models [15]. Its pharmacological effects involved tissue protection, anti-inflammatory and antioxidant effects, and mitochondrial protection [15,17,20]. These findings collectively suggest that purpurin possesses therapeutic potential for CIAKI as a candidate renoprotective agent.

After administration, ICM can accumulate extensively in renal tissue and induce pathological alterations, including renal tubular epithelial cell vacuolization, degeneration and necrosis, and tubular lumen narrowing [21,22]. The extent of renal injury in rats can be evaluated using indicators such as renal histopathological changes, the KW/BW ratio, and biochemical markers of renal function, notably the Scr and BUN levels [23]. In addition, ICM induce excessive ROS production through their direct cytotoxicity and indirect medullary hypoxia effects [24]. The overproduction of ROS not only induces DNA oxidation and lipid peroxidation but also activates the release of inflammatory cytokines such as TNF-α and IL-6 [25]. Collectively, this malignant cascade exacerbates damage to renal tissue, especially RTECs, promoting apoptosis [10]. In the present study, the pathological features in CIAKI model rats, such as decreased renal function, renal edema, oxidative stress, inflammatory responses, and mitochondrial dysfunction, confirmed the successful establishment of the model [26,27,28,29]. Notably, post-intervention with purpurin exhibits a clear dose-dependent protective effect. Specifically, administration of both low and high doses of purpurin significantly improved renal function parameters in model rats, alleviated renal edema and histopathological injury, and effectively suppressed oxidative stress and inflammatory levels. Early studies indicate that the tissue-protective effects of purpurin are primarily mediated through the activation of the Nrf2 pathway, ROS scavenging, and anti-inflammatory mechanisms, with these effects being particularly evident in neural and hepatic tissues [17]. Our results indicate that purpurin demonstrates potential therapeutic value for CIAKI.

ICM-induced mitochondrial ROS generation represents not only a pivotal event in the pathogenesis of CIAKI but also a promising therapeutic target. As mentioned, mitochondrial ROS generation and the release of pro-apoptotic factors can trigger apoptosis in renal tissue [6,7]. Guided by this observation, our study employed network pharmacology combined with bioinformatic analyses to determine the potential targets of purpurin against CIAKI, with the results revealing that it is closely associated with the anti-apoptotic PI3K/AKT pathway [30]. Molecular docking demonstrated high binding affinities between purpurin and apoptotic proteins, including PI3K, AKT1, ESR1, and SRC. Previous studies showed that PI3K/AKT activation can attenuate renal pathological progression in CIAKI animal models by inhibiting apoptosis or inducing autophagy [30]. Our in vivo experiments confirmed that purpurin effectively ameliorated the mitochondrial ultrastructure, particularly through reversing contrast-induced perturbations in mitophagy, thereby stabilizing mitochondrial function. In the context of CIAKI, this mitochondrial protective effect directly alleviates contrast agent-induced excessive ROS production and blocks initiation of the apoptotic cascade. In addition, purpurin significantly inhibits the Beclin1/Bcl-2/caspase-3 apoptotic cascade. In previous studies, it was found that Bcl-2 can directly interact with pro-apoptotic proteins Bax/Bak, or indirectly block the activation of caspase-3 by inhibiting the release of cytochrome C from mitochondria [26,27]. Therefore, the anti-apoptotic effect of purpurin may be attributed to its ability to upregulate the expression of the anti-apoptotic protein Bcl-2, and, additionally, to reduce mitochondrial ROS generation and stabilize mitochondrial function.

As an essential self-cleansing mechanism, autophagy enables cells to clear damaged organelles, misfolded proteins, and toxic metabolic byproducts, thereby maintaining cellular homeostasis [31]. Recent studies have identified that the activation of autophagy exerts a renoprotective effect in CIAKI cells and animal models [7,23,30]. Essentially, these studies provided evidence that the activation of autophagy contributes to reducing mitochondrial dysfunction and thus reduces oxidative stress, inflammatory responses, and cell apoptosis. In this study, purpurin significantly induced cell autophagy through upregulation of LC3-II expression and downregulation of p62 expression in the CIAKI model. LC3-II and p62 play a crucial role in autophagy. LC3-II is closely related to the formation and expansion of autophagosomes, which determine the cell components encapsulated and degraded by autophagosomes. P62 can specifically bind to LC3-II and promote its transport to autophagosomes for subsequent degradation [32,33,34,35]. Oxidative stress serves as one of the primary activation signals for autophagy. When intracellular levels of ROS increase, autophagy pathways are triggered to maintain cellular redox homeostasis by clearing damaged mitochondria and thus eliminating excess ROS. Therefore, exogenous induction of autophagy can effectively protect mitochondria and reduce mitochondrial ROS production, thereby exerting antioxidant effects [36]. In CI-AKI, contrast-induced oxidative stress leads to a short increase in LC3-II expression and thus triggers endogenous autophagy. However, the rapid elevation of ROS levels induces mitochondrial damage and dysfunction, which in turn impairs autophagic flux and promotes the accumulation of P62 and LC3-II, thereby exacerbating apoptosis and necrosis in renal tubular epithelial cells [7,37,38]. Combined with previous studies, enhancing the autophagy activity of renal tissue is a potential mechanism by which purpurin can mitigate ICM-induced kidney injury.

## 4. Materials and Methods

### 4.1. Network Pharmacology

#### 4.1.1. Prediction of Purpurin and CIAKI-Related Targets

Gene targets associated with purpurin and CIAKI were identified through the GeneCards database (https://www.genecards.org/ (accessed on 17 March 2025)), the DisGeNet database (https://disgenet.com/ (accessed on 17 March 2025)), and the SwissTargetPrediction database (http://swisstargetprediction.ch/ (accessed on 17 March 2025)).

#### 4.1.2. Construction of the Protein–Protein Interaction (PPI) Network

The common targets associated with purpurin and CIAKI were identified using Venny 2.1.0 (http://liuxiaoyuyuan.cn/ (accessed on 19 March 2025)) and then imported into the STRING database (https://cn.string-db.org (accessed on 19 March 2025)) with the following parameters: Organism: Homo sapiens; minimum interaction score: >0.4 (medium confidence). Genes with interaction scores above the threshold were identified as hub genes and were imported into Cytoscape v3.10.1 for visualization.

#### 4.1.3. Pathway Enrichment Analysis

The common targets were imported into the Metascape database (https://metascape.org/ (accessed on 23 March 2025)) for Gene Ontology (GO) analysis and Kyoto Encyclopedia of Genes and Genomes (KEGG) for pathway enrichment. The pathways were visualized using bioinformatics tools (https://www.bioinformatics.com.cn (accessed on 23 March 2025)). The visit time of the above network pharmacology-related website is April 2025.

### 4.2. Sequencing Analysis

The GSE189881 dataset was downloaded from the GEO database (NCBI GEO accession: GSE189881), which comprised RNA-Seq data from 3 control samples and 3 CIAKI model samples of SD rat kidneys. The raw data were preprocessed using the public platform of DAVID (https://davidbioinformatics.nih.gov/ (accessed on 2 April 2025)). Differentially expressed gene (DEG) analysis was subsequently performed to identify statistically significant differentially expressed genes.

### 4.3. Molecular Docking

The purpurin ligand molecule with a 2D structure was downloaded from the Pubchem database (https://pubchem.ncbi.nlm.nih.gov/ (accessed on 11 April 2025)). This small-molecule ligand was structured using Chem3D 14.0.0.17 software. The crystal structures of the target proteins were obtained from the RCSB Protein Data Bank (https://www.rcsb.org/ (accessed on 12 April 2025)). Structure preprocessing involved removing water molecules and original ligands using PyMOL 2.6.1 software. The molecular docking, binding energy calculation, docking result visualization, and analysis were performed using AutoDockTools 1.5.7 software. Maximum Grid Box was set to wrap the whole protein, and the protein domain was randomly docked. A binding energy of less than −5 kcal/mol is defined as good binding between the compound and the target protein.

### 4.4. Animals and Experimental Design

Thirty SPF-grade male Sprague-Dawley (SD) rats (6 weeks old, 200 ± 20 g; Animal Quality Certificate No. SCXK(YU) 2025-0005) were purchased from Henan Skobes Biotechnology Co., Ltd. and reared in the Animal Laboratory Center, Pharmacy School of Shihezi University. All procedures were approved by the Biology Ethics Committee of Shihezi University (Protocol No. A2025-706). Rats received food and water ad libitum before the experiment. After one week of acclimatization, rats were randomly assigned to five groups, with 6 rats in each group. The CIAKI modeling scheme was modeled on a previous study [28]. Briefly, rats underwent 48-h water deprivation, and furosemide (Veterinary medicine 041101181, Shanxi Zhaoyi Biological Co., Ltd., Yuncheng City, China) administration (15 mL/kg, i.m.) 0.5 h prior to iohexol (100 mL:35 g, Yangtze River Pharmaceutical Group, Taizhou City, China) administration (10 mL/kg, i.v.). After 24 h, those rats were orally administered saline (Model), 2.5 mg/kg purpurin (Shanghai Yuanye Bio-Technology Co., Ltd., B20974, Shanghai, China) (Model+LP), or 5 mg/kg purpurin (Model+HP), respectively. The control group received no treatment, and the HP group was orally administered 5 mg/kg purpurin. All rats were sacrificed by urethane (Shanghai Yuanye Bio-Technology Co., Ltd., S35916) anesthesia after 24 h of purpurin administration. Blood samples were collected via orbital blood collection and centrifuged at 3500× *g* for 15 min to obtain the serum. Kidney tissues were collected immediately at the end of the experiment, and were then stored at −80 °C or fixed in corresponding tissue fixation media.

### 4.5. Biochemical Analysis

#### 4.5.1. Assessments of Renal Function

Blood samples obtained from orbital blood collection were collected prior to water deprivation (day 0), at 24 h post-iohexol administration (day 3), and at 24 h post-purpurin/saline administration (day 4), respectively. The blood Scr (C011-2-1, Nanjing Jiancheng Bioengineering Institute, Nanjing, China) and BUN (C013-1-1, Nanjing Jiancheng Bioengineering Institute, Nanjing, China) levels of serum collected from each time-node were analyzed using corresponding commercial kits to determine kidney function.

#### 4.5.2. Assessments of Systemic Inflammation

The levels of tumor necrosis factor-alpha (TNF-α) (ml002859, Shanghai Enzyme-linked Biotechnology Co., Ltd., Shanghai, China), interleukin-1 beta (IL-1β) (ml003057, Shanghai Enzyme-linked Biotechnology Co., Ltd., Shanghai, China), and interleukin-6 (IL-6) (ml064292, Shanghai Enzyme-linked Biotechnology Co., Ltd., Shanghai, China) in serum collected at day 4 were detected using the double-antibody sandwich method of the enzyme-linked immunosorbent assay (ELISA). Samples were diluted 75 times and analyzed using the corresponding ELISA kits according to the manufacturer’s guidelines.

#### 4.5.3. Assessments of Renal Oxidative Stress

Renal tissues were homogenized in saline (1:9 *w*/*v*) using a bead mill homogenizer (4 °C, 60 Hz, 15 s grinding/30 s interval, 4 cycles) and centrifuged (4 °C, 3500 rpm, 10 min). The supernatants were used to analyze the malondialdehyde (MDA) (A003-1-2, Nanjing Jiancheng Bioengineering Institute, Nanjing, China) content, glutathione peroxidase (GSH-Px) (A005-1-2, Nanjing Jiancheng Bioengineering Institute, Nanjing, China) activity, superoxide dismutase (SOD) (A001-1-2, Nanjing Jiancheng Bioengineering Institute, Nanjing, China) activity, and nitric oxide (NO) (A013-2-1, Nanjing Jiancheng Bioengineering Institute, Nanjing, China) content according to manufacturers’ protocols from corresponding kits.

### 4.6. Histopathological Studies

#### 4.6.1. Hematoxylin and Eosin (HE) Staining

Kidney tissues were immersed in 8% polyformaldehyde for 48 h, followed by routine dehydration, transparentizing, and paraffin-embedding. Sections with a thickness of 5 μm were stained with HE reagents and observed under a microscope (Microsystems CMS GmbH DM3000 LED, Leica, Wetzlar, Germany). The tubular injury was scored using the Paller scoring system [39].

Tubular necrosis and proteinaceous casts were graded as follows: 0 = no damage, 1 = mild (unicellular, patchy, isolated damage), 2 = moderate (<25% damage), 3 = severe (25–50% damage), or 4 = very severe (>50% damage).

#### 4.6.2. Terminal Deoxynucleotidyl Transferase dUTPnick-End Labeling (TUNEL) Staining

The paraffin-embedded tissue sections (4 μm) were detected in the apoptotic cells using the TUNEL kit (E-CK-A320, Elabscience Biotechnology Co., Ltd., Wuhan, China). Briefly, sections were immersed with a mixture of TdT Equilibration Buffer, Labeling Solution, and TdT Enzyme (35:10:5) for 1 h at 37 °C and incubated with DAPI for 5 min at room temperature without light; then, they were observed and photographed under a microscope for analysis.

#### 4.6.3. Transmission Electron Microscopy

Fresh renal cortical tissues (1 mm^3^) were fixed in 3% glutaraldehyde (dissolved in 0.1 M phosphate buffer, pH 7.4) for 12 h at 4 °C, followed by dehydration using a graded series of ethanol and embedding using acetone and epoxy resin overnight. Sections with a thickness of 70 nm were observed under a transmission electron microscope (H-7650, Hitachi, Tokyo, Japan).

### 4.7. Western Blot Analysis

Renal tissue lysates were prepared with RIPA (G2002; ServiceBio, Wuhan, China) buffer and quantified using a bicinchoninic acid assay. Protein samples were separated by 8% SDS-PAGE, and the electrophoresis conditions were set as 80 V in concentrated gel and 100 V in separation gel. After electrophoresis, the protein was transferred from the gel to the PVDF membrane at 300 mA in an ice bath. After blocking with 5% skim milk in TBST for 1.5 h at room temperature, membranes were incubated overnight at 4 °C with the following primary antibodies: Anti-Beclin 1 (1:1500, CY5092; Abways, Shanghai, China), Anti-BAX (1:1000, CY5059; Abways, Shanghai, China), Anti-Bcl-2 (1:1000, CY5032; Abways, Shanghai, China), Anti-Caspase 3 (1:1000, CY5051; Abways, Shanghai, China), Anti-Caspase 8 (1:1000, CY5584; Abways, Shanghai, China), Anti-p62 (1:500, GB11531-100; ServiceBio, Wuhan, China), and Anti-LC3B (1:500, GB113801-100; ServiceBio, Wuhan, China). Membranes were subsequently incubated with HRP-conjugated Goat Anti-Rabbit IgG secondary antibody (1:10,000, AB0101; Abways, Shanghai, China) for 2 h at room temperature. Protein bands were visualized using enhanced chemiluminescence substrate, and quantitative analysis of band intensities was performed using ImageJ 1.54p software.

### 4.8. Statistical Analysis

Statistical analyses were performed using GraphPad Prism software (version 8.0.2). Normally distributed data are presented as mean ± standard deviation (SD). A Shapiro–Wilk test was performed after data collection to verify the rationality of the normal distribution of the array (*p* > 0.05 for each group). Subsequently, one-way analysis of variance (ANOVA) was performed; *p* < 0.05 was considered statistically significant.

## 5. Conclusions

This study demonstrates that purpurin, a natural anthraquinone, significantly ameliorates CIAKI rat models in a dose-dependent manner. Its nephroprotective effects are evidenced by the attenuation of oxidative stress, inflammation, and cellular apoptosis. Mechanistically, purpurin potentially exerts its effects by modulating the Beclin1/Bcl-2/caspase-3 and P62/LC3 pathways, thereby effectively stabilizing mitochondrial function and enhancing autophagic activity to counteract apoptosis driven by oxidative stress and inflammation. This work provides evidence that purpurin exerts antioxidant and renal protection effects by inducing autophagy and suppressing apoptosis, offering a novel candidate compound. Given the broad therapeutic potential of autophagy inducers, the upstream mechanism by which purpurin activates autophagy warrants further investigation in order to provide a theoretical foundation for future clinical translation.

## Data Availability

The raw data supporting the conclusions of this article will be made available by the authors upon request.

## Abbreviations

The following abbreviations are used in this manuscript:

CIAKI	Contrast-induced acute kidney injury
ICM	Directory of open access journals iodinated contrast media
RTECs	Renal tubular epithelial cells

## References

[1] Interventional Cardiology Group, Chinese Society of Cardiology, Chinese Medical Association, Macrovascular Group, Chinese Society of Cardiology, Chinese Medical Association, Editorial Board of Chinese Journal of Cardiology (2021). Chinese expert consensus on management strategies for adverse events related to intra-arterial use of iodine contrast media during cardiovascular intervention. Zhonghua Xin Xue Guan Bing Za Zhi.

[2] Kusirisin P., Chattipakorn S.C., Chattipakorn N. (2020). Contrast-induced nephropathy and oxidative stress: Mechanistic insights for better interventional approaches. J. Transl. Med..

[3] Wang H., Lambourg E., Guthrie B., Morales D.R., Donnan P.T., Bell S. (2022). Patient outcomes following AKI and AKD: A population-based cohort study. BMC Med..

[4] van der Molen A.J., Krabbe J.G., Dekkers I.A., Geenen R.W.F., Bellin M.-F., Bertolotto M., Brismar T.B., Cadamuro J., Correas J.-M., Heinz-Peer G. (2024). Analytical interference of intravascular contrast agents with clinical laboratory tests: A joint guideline by the ESUR Contrast Media Safety Committee and the Preanalytical Phase Working Group of the EFLM Science Committee. Clin. Chem. Lab. Med..

[5] Owen R.J., Hiremath S., Myers A., Fraser-Hill M., Barrett B.J. (2014). Canadian Association of Radiologists consensus guidelines for the prevention of contrast-induced nephropathy: Update 2012. Can. Assoc. Radiol. J..

[6] Cashion W., Weisbord S.D. (2022). Radiographic Contrast Media and the Kidney. Clin. J. Am. Soc. Nephrol.

[7] Lin Q., Li S., Jiang N., Jin H., Shao X., Zhu X., Wu J., Zhang M., Zhang Z., Shen J. (2021). Inhibiting NLRP3 inflammasome attenuates apoptosis in contrast-induced acute kidney injury through the upregulation of HIF1A and BNIP3-mediated mitophagy. Autophagy.

[8] Oh H., You J.S., Bae H., Bin Park G., Chung Y.E. (2023). Delivery of recombinant sestrin2 ameliorates oxidative stress, mitochondrial damage and renal dysfunction in contrast-induced acute kidney injury. Biochem. Pharmacol..

[9] Zhou L.-Y., Liu K., Yin W.-J., Xie Y.-L., Wang J.-L., Zuo S.-R., Tang Z.-Y., Wu Y.-F., Zuo X.-C. (2023). Arginase2 mediates contrast-induced acute kidney injury via facilitating nitrosative stress in tubular cells. Redox Biol..

[10] Zhang C., Suo M., Liu L., Qi Y., Zhang C., Xie L., Zheng X., Ma C., Li J., Yang J. (2021). Melatonin Alleviates Contrast-Induced Acute Kidney Injury by Activation of Sirt3. Oxidative Med. Cell. Longev..

[11] Fähling M., Seeliger E., Patzak A., Persson P.B. (2017). Understanding and preventing contrast-induced acute kidney injury. Nat. Rev. Nephrol..

[12] Gao T., Gu R., Wang H., Li L., Zhang B., Hu J., Tian Q., Chang R., Zhang R., Zheng G. (2024). The Protective Role of Intermedin in Contrast-Induced Acute Kidney Injury: Enhancing Peritubular Capillary Endothelial Cell Adhesion and Integrity Through the cAMP/Rac1 Pathway. Int. J. Mol. Sci..

[13] Peng L., Luo Y., Tan F., Chen Q., Wang J., Ouyang X., Wu B., Tang X., Li S. (2025). microRNA-30c attenuates contrast-induced acute kidney injury by reducing renal tubular epithelial cell apoptosis via targeting SOCS1. Exp. Cell Res..

[14] Lin Y., Lin J., Guo Y., Li X., Tan X., Long M., Li Y., Liao X., Huang Z. (2020). Atorvastatin protects against contrast-induced acute kidney injury via upregulation of endogenous hydrogen sulfide. Ren. Fail..

[15] Singh J., Hussain Y., Luqman S., Meena A. (2021). Purpurin: A natural anthraquinone with multifaceted pharmacological activities. Phytother. Res. PTR.

[16] Nam W., Kim S.P., Nam S.H., Friedman M. (2017). Structure-Antioxidative and Anti-Inflammatory Activity Relationships of Purpurin and Related Anthraquinones in Chemical and Cell Assays. Molecules.

[17] Ren Q., Bakker W., Wesseling S., Bouwmeester H., Rietjens I.M.C.M. (2023). On the Role of ROS and Glutathione in the Mode of Action Underlying Nrf2 Activation by the Hydroxyanthraquinone Purpurin. Antioxidants.

[18] Ingelmo A.R., Diaz I.D., Moreno R.C., Quesada M.C.M., García-Avilés C., Nuñez I.G., Tadeo J.I.M., Ballesteros R.M., Ortega-Rodríguez N., Vilchez M.A.P. (2016). Clinical Practice Guidelines for Diagnosis and Management of Hypersensitivity Reactions to Contrast Media. J. Investig. Allergol. Clin. Immunol..

[19] Perazella M.A., Rosner M.H. (2022). Drug-Induced Acute Kidney Injury. Clin. J. Am. Soc. Nephrol..

[20] Hussain Y., Singh J., Raza W., Meena A., Rajak S., Sinha R.A., Luqman S. (2022). Purpurin ameliorates alcohol-induced hepatotoxicity by reducing ROS generation and promoting Nrf2 expression. Life Sci..

[21] Lakhal K., Ehrmann S., Robert-Edan V. (2020). Iodinated contrast medium: Is there a re(n)al problem? A clinical vignette-based review. Crit. Care.

[22] Wu T., Zhu W., Duan R., Sun J., Bao S., Chen K., Han B., Chen Y., Lu Y. (2024). Magnetic vagus nerve stimulation ameliorates contrast-induced acute kidney injury by circulating plasma exosomal miR-365-3p. J. Nanobiotechnol..

[23] Zuo Z., Li Q., Zhou S., Yu R., Wu C., Chen J., Xiao Y., Chen H., Song J., Pan Y. (2024). Berberine ameliorates contrast-induced acute kidney injury by regulating HDAC4-FoxO3a axis-induced autophagy: In Vivo and In Vitro. Phytother. Res. PTR.

[24] Heyman S.N., Rosen S., Khamaisi M., Idée J.-M., Rosenberger C. (2010). Reactive oxygen species and the pathogenesis of radiocontrast-induced nephropathy. Investig. Radiol..

[25] Peng L., Li S., Huang Q., Sun Y., Sun J., Luo T., Wang Y., Hu Z., Lai W., Peng H. (2025). Irisin-mediated muscle-renal crosstalk as a protective mechanism against contrast-induced acute kidney injury via cGAS-STING signalling inhibition. Clin. Transl. Med..

[26] Wang X., Luo T., Yang Y., Yang L., Liu M., Zou Q., Wang D., Yang C., Xue Q., Liu S. (2024). TRPA1 protects against contrast-induced renal tubular injury by preserving mitochondrial dynamics via the AMPK/DRP1 pathway. Free Radic. Biol. Med..

[27] Song Z., Li J., Gong X. (2025). Dahuang chuanxiong decoction against contrast-induced nephropathy: Multi-omics, crosstalk between BNIP3-mediated mitophagy and IL-17 pathway. Phytomed. Int. J. Phytother. Phytopharm..

[28] Lin Y., Zhu G., Li X., Yu H., Luo Y., Lin J., Li R., Huang Z. (2022). Icariin and Competing Endogenous RNA Network: A Potential Protective Strategy Against Contrast-Induced Acute Kidney Injury. Drug Des. Dev. Ther..

[29] Chen W., Lu H., Dai W., Li H., Chen Y., Liu G., He L. (2025). PUM2 Lowers HDAC9 mRNA Stability to Improve Contrast-Induced Acute Kidney Injury through Attenuating Oxidative Stress and Promoting Autophagy. Diabetes Metab. J..

[30] Lin Q., Li S., Jiang N., Shao X., Zhang M., Jin H., Zhang Z., Shen J., Zhou Y., Zhou W. (2019). PINK1-parkin pathway of mitophagy protects against contrast-induced acute kidney injury via decreasing mitochondrial ROS and NLRP3 inflammasome activation. Redox Biol..

[31] Kim D.-H., Park J.S., Choi H.-I., Kim C.S., Bae E.H., Ma S.K., Kim S.W. (2021). The critical role of FXR is associated with the regulation of autophagy and apoptosis in the progression of AKI to CKD. Cell Death Dis..

[32] Hwang H.J., Ha H., Lee B.S., Kim B.H., Song H.K., Kim Y.K. (2022). LC3B is an RNA-binding protein to trigger rapid mRNA degradation during autophagy. Nat. Commun..

[33] Zheng J., Wei S., Xiao T., Li G. (2021). LC3B/p62-mediated mitophagy protects A549 cells from resveratrol-induced apoptosis. Life Sci..

[34] Omrane M., Ben M’bArek K., Santinho A., Nguyen N., Nag S., Melia T.J., Thiam A.R. (2023). LC3B is lipidated to large lipid droplets during prolonged starvation for noncanonical autophagy. Dev. Cell.

[35] Huang X., Zhang J., Yao J., Mi N., Yang A. (2025). Phase separation of p62: Roles and regulations in autophagy. Trends Cell Biol..

[36] Su L., Zhang J., Gomez H., A Kellum J., Peng Z. (2023). Mitochondria ROS and mitophagy in acute kidney injury. Autophagy.

[37] Yang X., Yan X., Yang D., Zhou J., Song J., Yang D. (2018). Rapamycin attenuates mitochondrial injury and renal tubular cell apoptosis in experimental contrast-induced acute kidney injury in rats. Biosci. Rep..

[38] Zhu X., Lin Q., Yang Y., Li S., Shao X., Zhang W., Cai H., Li J., Wu J., Zhang K. (2024). αKlotho modulates BNIP3-mediated mitophagy by regulating FoxO3 to decrease mitochondrial ROS and apoptosis in contrast-induced acute kidney injury. Cell. Mol. Life Sci..

[39] Chang Y., Han Z., Zhang Y., Zhou Y., Feng Z., Chen L., Li X., Li L., Si J.-Q. (2019). G protein-coupled estrogen receptor activation improves contractile and diastolic functions in rat renal interlobular artery to protect against renal ischemia reperfusion injury. Biomed. Pharmacother..

